# Electronic Nose Feature Extraction Methods: A Review

**DOI:** 10.3390/s151127804

**Published:** 2015-11-02

**Authors:** Jia Yan, Xiuzhen Guo, Shukai Duan, Pengfei Jia, Lidan Wang, Chao Peng, Songlin Zhang

**Affiliations:** College of Electronics and Information Engineering, Southwest University, Chongqing 400715, China; E-Mails: yanjia119@163.com (J.Y.); swugxz@163.com (X.G.); jiapengfei200609@126.com (P.J.); ldwang@swu.edu.cn (L.W.); pengchaocg@163.com (C.P.); z574066616@163.com (S.Z.)

**Keywords:** electronic nose, feature extraction methods, review

## Abstract

Many research groups in academia and industry are focusing on the performance improvement of electronic nose (E-nose) systems mainly involving three optimizations, which are sensitive material selection and sensor array optimization, enhanced feature extraction methods and pattern recognition method selection. For a specific application, the feature extraction method is a basic part of these three optimizations and a key point in E-nose system performance improvement. The aim of a feature extraction method is to extract robust information from the sensor response with less redundancy to ensure the effectiveness of the subsequent pattern recognition algorithm. Many kinds of feature extraction methods have been used in E-nose applications, such as extraction from the original response curves, curve fitting parameters, transform domains, phase space (PS) and dynamic moments (DM), parallel factor analysis (PARAFAC), energy vector (EV), power density spectrum (PSD), window time slicing (WTS) and moving window time slicing (MWTS), moving window function capture (MWFC), *etc*. The object of this review is to provide a summary of the various feature extraction methods used in E-noses in recent years, as well as to give some suggestions and new inspiration to propose more effective feature extraction methods for the development of E-nose technology.

## 1. Introduction

An electronic nose (E-nose) is an instrument which comprises an array of electronic chemical sensors with partial specificity and an appropriate pattern-recognition system, capable of recognising simple or complex odors. It is specifically used to sense odorant molecules in analogy to the human nose. However, the architecture of an E-nose also applies in gas sensing for the detection of individual components or mixtures of gases/vapours [1], which is playing an increasing role in general purpose detection of gases in many applications such as odor analysis [2,3,4], quality control of food industry [5,6,7,8,9,10], environment protection [11,12,13], public health [14,15,16,17,18,19], explosives detection [20] and spaceflight applications [21]. The main hardware component of an E-nose is an array of non-specific gas sensors, *i.e.*, sensors that interact with a broad range of chemicals with varying strengths. Correspondingly, the analyte stimulates sensors in the array, which elicits a characteristic response called “fingerprint”. The main software component of an E-nose is its feature extraction and pattern recognition algorithms, which process the sensor characteristic response, extract and select useful information and realize the pattern recognition.

According to the components of an E-nose system, the performance improvement of an E-nose system mainly contains three kinds of optimization: sensitive material selection and sensor array optimization, feature extraction and selection method, and pattern recognition method. Many research groups are focusing on improving the performance of E-noses [22,23,24]. In fact, they mainly emphasize the three types of optimizations. Sensitive material selection and sensor array optimization refers to the hardware structure of the E-nose. If the E-nose is homemade, a more typical approach is to first research the chemical composition of the samples and then select sensors which are responsive to those chemical groups with cross sensitivity to form the sensor array. Then from this, you would tune and adjust the sensors in the array according to the responses, which is called sensitive material selection and sensor array optimization. The optimization is all-round, as the type of sensors, the number of sensors, even the position, order and topological structure of sensors *etc.*, can affect the kinetic processes of the sensor response. However, if a commercial E-nose is used, we just get what is in the array and cannot change the hardware structure of the E-nose. This means that we cannot perform sensor array optimization from the hardware perspective. If we wish to improve the performance of an E-nose we must emphasize the feature extraction and selection method as well as the pattern recognition method. Although performance improvement of an homemade E-nose system can be done through sensitive material selection and sensor array optimization, the feature extraction method is one of the key points of performance improvement of E-nose systems because feature extraction is the first step of the sensor signal processing and the feature selection [25] and pattern recognition are performed on the basis of features which play a significant role in affecting the effect of the subsequent feature selection and pattern recognition algorithms. No matter which pattern recognition method is been selected its recognition rate has a strong relationship with the sample distribution in the feature space. The aim of feature extraction is to extract robust information from the characteristic sensor response with less redundancy, which can represent the different “fingerprint” patterns well, to ensure the effectiveness of the subsequent pattern recognition algorithm. However, it is still a great challenge for people to extract features from the “fingerprint” to further improve the pattern recognition accuracy.

According to the research on feature extraction during the most recent twenty years, there are many feature extraction methods which have been used in E-nose applications. Most of these feature extraction methods can be roughly divided into three types: the first type is to extract piecemeal signal features [26] from the original response curves of sensors, such as maximum values, integrals, differences, primary derivatives, secondary derivatives, the adsorption slope, and the maximum adsorption slope at a specific interval from the response curves, *etc.* The second type is based on curve fitting [27], which fits the response curves based on a specific model and extracts a set of fitting parameters as the features. The third one is based on some transforms, and very often the fast Fourier transform (FFT) [28,29,30,31] and the discrete wavelet transform (DWT) have been used as feature extraction tools for this purpose [6,28,32].

Besides the above conventional feature extraction methods, many new original methods have been proposed in recent years. Phase space (PS) [33] and dynamic moments (DM) [34], which are usually used in dynamical systems, are also applied to E-nose signals for extraction. Parallel factor analysis (PARAFAC) [35,36] is one of the most popular multi-way data decomposition methods and is attracting increasing interest because it is a processing technique that simultaneously determines the pure contributions to the dataset, optimizing each factor as a time, in trilinear systems. Energy vector (EV) [37] is a vector of energy, which contains the energy of each sensor and all the mutual energies. It is suitable to study the relations between signals of sensors belonging to the same array. In addition, the power density spectrum (PSD) method [38] is also applied in E-nose feature extraction. Another type of methods are based of window functions, especially windowed time slicing (WTS) [39,40,41], wherein the time response of each sensor is multiplied by time windows to obtain the area values and these values are further used as features.

This review provides a summary of the main methods of feature extraction used in E-noses in recently years, which are conducive to analysis and research on E-nose technology, by describing and comparing the basic types of feature extraction methods which differ as the application and E-nose experiments change and by providing examples of research in which E-noses have been utilized to analyze and detect different materials. In addition, we also hope that more potential and effective feature extraction methods can be proposed in further work to enhance the performance of E-noses with the inspirations proposed in this paper.

## 2. Feature Extraction Methods

### 2.1. Feature Extraction from Original Response Curves

The first feature extraction method is to extract piecemeal signal features from the original response curves of sensors, including steady-state response and transient responses such as maximum value, integrals, derivatives, area values, rising time, falling time, rising slopes, falling slopes, *etc*. Because the maximum value represents the final steady-state feature of the entire dynamic response process in the final balance, which reflects the maximum reaction degree change of sensors responding to odors, it is usually used as the most common and simple E-nose feature. In the early stages of the published history of E-noses, transient information was not used in sensor signal feature extraction. A variety of steady-state models (maximum value) have been used as feature extraction method for gas sensor signals, as illustrated in Table 1.

**Table 1:** Some maximum value feature models.

Model	Description	References
Difference	$x_{ij}=V_{ij}^{\max}-V_{ij}^{\min}$	[42]
Relative difference	$x_{ij}=V_{ij}^{\max}/V_{ij}^{\min}$	[43]
Fractional difference	$x_{ij}=\left(V_{ij}^{\max}-V_{ij}^{\min}\right)/V_{ij}^{\min}$	[44]
Logarithm difference	$\log\left(V_{ij}^{\max}/V_{ij}^{\min}\right)$	[45]
Sensor normalization	$x_{ij}=x_{ij}/\left(x_{i}^{\max}-x_{i}^{\min}\right)$	[45,46]
Array normalization	$x_{ij}=x_{ij}/\sum_{i}x_{ij}^{2}$	[47,48]

$x_{ij}$ and $V_{ij}^{\max}$ represent the processed maximum value feature and the original maximum response value of the of the *i-*th sensor to the *j*-th gas, respectively. The difference method can usually eliminate the additive errors, which are added both to the baseline and the steady-state response (meaning the response of the *i*-th sensor to the *j*-th gas). There is some evidence that the relative and fractional difference are helpful to compensate for the temperature influence on the sensors and the fractional difference linearizes the mechanism that generates a concentration dependence in metal oxide chemiresistor sensors [47]. The log difference is more suitable when the variation of the concentration of the odor is very large because it is able to linearize the highly nonlinear relationship between odor concentration and the sensor output. The normalization models, which limit each sensor output between 0 and 1, thereby keep each element of the response vector at the same magnitude. Not only can it reduce the calculation error of stoichiometric recognition, but also is very effective when it is not the odor concentration which is of interest, but rather the precise identification of the odor. This method for example provides good recognition results for the discrimination of several types of coffee using neural networks [48].

Besides the steady-state features of response curves, various other transient features such as derivatives and integrals of original response curves were taken into research in many special applications. Balasubramanian *et al*. used a MOS-based E-nose system, which was designed and fabricated at the Bio-Imaging and Sensing Center, North Dakota State University (Fargo, ND, USA) to analyze the headspace from beef strip loins stored at 4 °C and 10 °C. The designed system consisted of a cylindrical sampling chamber made of Teflon on to which seven thick film MOS (Figaro USA Inc., Glenview, IL, USA) and one integrated sensor sensitive to temperature and relative humidity (Sensirion AG, Zurich, Switzerland) were mounted at regular intervals. The headspace gas from the meat packages was drawn into the chamber using a diaphragm-type air pump, and a fan and valve assembly fixed at the bottom of the sample chamber facilitated purging the sensor array after analysis. Six areas features under the response curves were extracted from six gas sensors (TGS 812, TGS 822, TGS 880, TGS 2602, TGS 2611-1 and TGS 2611-2). Another four features were extracted from the remaining two sensors related to the relative humidity (RH), temperature and carbon dioxide readings. The results showed that the MOS-based electronic system integrated with radial basis function neural networks (RBFNN) obtained above 90% total maximum classification accuracy in identifying spoiled meat samples from the unspoiled meat samples with six area features and four additional environmental features for the meat samples stored at 4 °C and 10 °C [49].

Distante *et al.*, used a homemade E-nose consisting of five SnO_2_ sensors prepared by means of sol-gel technology with Pd, Pt, Os, and Ni as doping elements. The film thickness was 100 nm and the films were deposited on alumina substrates supplied with interdigitated electrodes and platinum heater, by the spin coating technique at 3000 rpm, dried at 80 °C and heat treated in air at 600 °C. After deposition, the sensors were mounted onto a TO8 socket and inserted in a test chamber. Three volatile organic compounds (VOCs) were investigated: acetone, hexanal and pentanone in 50% relative humidity (RH) and dry air. Several feature extraction methods, both steady-state feature maximum value and transient feature, were considered. The results showed that integral and derivatives methods have higher recognition rates than that of maximum values and they also concluded that the desorption stage is more informative than the adsorption stage of the original response curves [50].

Roussel *et al.*, applied a homemade E-nose to discriminate satisfactory wines and unsatisfactory vinegar flavour wines. The measurement device is a metal oxide gas sensor array containing five Figaro TGS series SnO_2_ sensors (TGS 800, 822, 824, 825 and 842) and modified to host a thermo-hygrometer and to acquire the sensor voltage on a 12 bit A/D converter. Twenty-nine features are extracted from each sensor response curve including seven mean response values on different time intervals, six slopes on different time intervals, eight primary derivatives and eight secondary derivatives. And then these features have been validated and evaluated qualitatively by three specific indexes: the repeatability, discrimination and redundancy. However, the authors just proposed the three indexes to qualitatively analyze the features but did not give any classification results for specific data using different features, which were considered better than others according to their proposed three evaluating indexes [51].

Llobet *et al*. established an array to discriminate between and quantify ethanol, toluene and *o*-xylene at concentration ranges from 25 ppm to 100 ppm, which consisted of four commercially available Taguchi gas sensors: two TGS822, one TGS813 and one TGS800. The model TGS822 exhibits high sensitivity to alcohol and organic solvents. The model TGS813 is mainly sensitive to general combustible gases and the model TGS800 is sensitive to gasoline exhaust and other air contaminants. The steady-state feature (conductance change ΔG) and conductance rise time (Tr, measured from 20% to 60% of ΔG) were extracted as features and they found that the using Tr as feature gave much better qualitative recognition results and using ΔG and Tr as features results in equal quantitative recognition rates, as shown in Table 2, which meant that Tr was concentration-independent [26].

**Table 2:** Classification rates (CR) of different features in [26].

Features	Qualitative CR (%)	Quantitative CR (%)
Difference maximum value ΔG	66.7	95
Conductance rise time Tr	92	95

Eklöv *et* *al.*, investigated the concentration of gas mixtures of hydrogen and ethanol with a homemade E-nose with four platinum MOSFET sensors, which had a thin discontinuous platinum film covered gate oxide. The gate voltages of each MOSFET sensor were detected and the sampling frequency was set at 1 Hz. All measurements consisted of an absorption stage for 2 min and a desorption stage for 8 min with a flow rate of 100 mL/min. A sequence of different concentrations of hydrogen and ethanol that ranged between 0 and 50 ppm were tested. The extracted features could be divided into two types: simple parameters from the original response curves and coefficients from different curve fitting models. It is necessary to select these parameters systematically and take into consideration the noise properties as well as the relationship between the features and the recognition ability of the features in a specific problem [52]. The features extracted from original response curves are shown in Table 3. Several combinations of features were proposed and gave satisfactory concentration prediction results.

**Table 3:** Description of features extracted from original response curves in [52].

Parameters	Description
Baseline	$\frac{1}{5}\sum_{T=\mathrm{gason}-4\text{s}}^{\mathrm{gasOn}}\text{(sensor value)}$
Final response, response	Sensor value (averaged over 5 s) at gasOff−baseline
30/90 s on/off response	Sensor value (averaged over 5 s) 30/90 s after gasOn/Off—baseline
Maximum response	Max(sensor value)—baseline
Min/max derivative	Min/max difference between two samples during measurement
On/off derivative	(Sensor value 10 s after gasOn/Off−baseline)/10
Plateau derivative	(Response−90 s on response)/30
Derivative	$\sum_{T＝\mathrm{gason}}^{\mathrm{gasOff}}\text{(sensor value}-\text{baseline)}$
Off integral	$\sum_{T＝\mathrm{gasoff}}^{\mathrm{gasOff}+119\text{s}}\text{(sensor value}-\text{baseline)}$
Short on/off integral	$\sum_{T=\mathrm{gason/gasoff}}^{\mathrm{gasOn}/\mathrm{gasoff}+9\text{s}}\text{(sensor value}-\text{baseline)}$
Response/on integral	Response/on integral
T0-90%	Time from gasOn for sensor value to reach baseline + 0.9 × response
T0-60%	Time from gasOn for sensor value to reach baseline + 0.6 × response
T100-10%	Time from gasOff for sensor value to reach baseline + 0.1 × response
T100-40%	Time from gasOff for sensor value to reach baseline + 0.4 × response

These parameters can be assessed according to the signal to noise ratio (SNR), correlation and significance in a 2-dimensional PCA plot. The signal to standard deviation ratio (S/σ-ration) [52], defined in Equation (1), was used to estimate the property of the features. It can provide similar information as a SNR:

(1) $$S/\sigma -ration=\frac{{\text{stdev}}_{\text{all concentration steps}}({\text{average}}_{\text{all measurments at each concentration step}}(feature))}{{\text{stdev}}_{\text{all concentration steps}}(feature-{\text{average}}_{\text{all measurments at this concentration step}}(feature))}$$

In addition, principal component analysis (PCA) was used to analyze the features. The PCA result gave score plots and loading plots. The trends, groups and outliers of the samples can be observed in score plots and the correlation and similarity between the features can be observed. A longer distance from the origin in the loading plot and a larger SNR implied that the feature contained more information. The artificial neural networks (ANN) classification results showed that: (1) a large error is obtained only with the final response features; (2) it was possible to improve the prediction accuracy to use features according to the S/σ-ration calculation; (3) there was information which can improve the recognition ability in the loading plot; (4) features extracted from original response curves may gave comparable results as the complex curve fitting coefficients; (5) there was more information about the detected materials in the shape of the whole response than only the response value.

Zhang *et al*. used an array of six TGS gas sensors to detect four common flammable liquids (gasoline, kerosene, diesel oil and ethanol) and three incombustible drinks (ice black tea, orange juice and Coca Cola) in less than 10 s for each recognition. Because they needed to realize fast recognition with an E-nose, they must extract robust information from the first few seconds of the sensor response curves as less redundant as possible. They researched four features (integrals, differences, primary derivatives and secondary derivatives) at a specific interval from the original response curves to classification [53]. Because integrals, differences, primary derivatives and secondary derivatives of the response curves are continuous with the interval changes, different positions of the curves can contain different information, which may consecutively change with the interval changes. Therefore, a calculating process was applied to fix at which interval of the response curves robust information can be extracted *S_t_* shown in Equation (2), was use to evaluated the information quality of the feature extracted at the time *t* of the response curves from the sensor array, which is a criterion to measure the separative degree among different classes of data based on the scatter matrix [54]. The expression of *S_t_* is:

(2) $$S_{t}=T_{r}\{\text{S}{\text{w}}_{t}^{-1}\text{S}{\text{m}}_{t}\}$$

where Sw*_t_* is the scatter matrix within a class, while $\text{S}{\text{m}}_{t}$ is the scatter matrix among different classes. PCA and discriminant function analysis (DFA) results showed that 85.7% classification rate was obtained with the four features in optimal time *t*, while only 57.1% classification rate was obtained with the maximum value feature.

Šetkus *et al.*, applied a homemade E-nose with s metal oxide (MOX) sensors array that contained commercial- and laboratory-made sensors to investigate some volatile organic compounds (VOCs), namely acetone, acetic acid, butyric acid, and acetaldehyde. The sensor array included two TGS type sensors (TGS2602, TGS2610). The laboratory-made sensors were based on tin oxide thin films, which were modified by catalytic metals such as Au, Pt, Pd and Ru, and were grown by rheotaxial (fused metal) growth and the thermal oxidation technique (RGTO). The authors researched three kinetic features: (1) the time the maximum value (τ_max_); (2) the time interval between the beginning of the adsorption and the end of the transient signal; (3) the slope of the response calculated in the desorption stage. PCA results showed that the dynamic features added significant information and allowed a better discrimination of the VOCs than that of the relative maximum response value [55].

**Table 4:** Description of features extracted from original response curves in [56].

Extracted Feature	Represent Meaning
Max slope	The respond rate of sensor to different vinegar gas
Maximum	The maximum respond value
Average of last 20 points	The stationary phase of equilibrium between reversible adsorption and desorption
Average of whole points	Sensor respond value during the whole process

Zou *et al.*, used an E-nose based on five tin-oxide sensors from Figaro Co. Ltd. (TGS 813, TGS 880, TGS 822, TGS 825, TGS 812), one humidity sensor (HS-01) and one temperature sensor (Pt100) to identify two different types of vinegars. Four features are extracted from each curve, which are shown in Table 4. They are the slope max, maximum, average of the last 20 points and the average of whole points of curve [56]. In addition, an evaluation index, called “distinguish index” (DI), was proposed to evaluate the features.

Wei *et al.*, extracted the maximum values, area values and 70th s values as the features from the E-nose responses and the PCA results indicated that the 70th s values provided the most accurate results when classifying unshelled peanuts and peanut kernels, which could be distinguished reliably by using a two-dimensional PCA [57].

From the above, many features can be extracted from original response curves (Table 5). Some of the features have specific physical meanings and reflect different information of the reaction kinetics at different aspects. Maximum value represents the final steady-state feature of the entire dynamic response process in the final balance, reflecting the maximum reaction degree change of sensors responding to odors. Intervals may represent the accumulative total of the reaction degree changing and differences may represent the degree of the reaction. Primary derivatives (slopes) may represent the rate of the reaction of sensors responding to odors and secondary derivatives may represent the acceleration of the reaction, *etc*. [53,58].

**Table 5:** Summary of commonly used features extracted from original response curves

Feature	Description	References
Maximum response	Max(sensor value)	[42,43,44,45,46,47,51,52,55,56,59]
Responses of special time	Response value of special time in the whole response curve	[51,52,56,57]
Time of special responses	Time of special response value in the whole response curve	[26,51,52,55]
Area	Area values of sensor response curve and time axis surrounded	[49,57]
Integral	$I=\int_{\text{a}}^{b}x(t)dt$	[50,52,53,59]
Derivative	$D\prime =\frac{dx(t)}{dt}$	[50,51,52,53,56]
Difference	$x(t_{j})-x(t_{i})$	[52,53,55,59]
Second derivative	$D\prime \prime =\frac{d^{2}x(t)}{d^{2}t}$	[51,53]

By extracting these features we can get information about different aspects from the original reactions between the sensors and the odors. However, it is evident that which extraction methods from the original response curves are appropriate depends on the different sensors and applications and for different applications and sensors, the feature extraction methods which give the best features are different. Moreover, researchers are likely to use composite features because these types of features which usually contain steady-state and transient features can obtain better performance. It is not easy to draw a conclusion about which method is the best one. On the whole, for maximum features, the normalization models give better performance than the other models. The transient features can receive better recognition accuracy than that of the steady-state feature and especially integral methods generally give the best features for many different E-nose applications. Based on the previous researches, a summary is listed in Table 5, which provides a brief description of the features commonly used in previous researches.

### 2.2. Features Extraction from Curve Fitting Parameters

Instead of extracting characteristics from the raw data curve, an alternative method is to model sensor response curves and then extract features based on the model coefficients. Curve fitting is a data processing method which approximately characterizes or analogizes the functional relationship of discrete points using continuous curves. The curve fitting method approximates discrete data using analytical expressions. The basic idea is to determine the fitting function in accordance with the trend of the discrete points. It utilizes the discrete points, but is not limited to these discrete points. In curve fitting, the critical and complex problem is how to select and envision the specific form of the unknown function.

There are two strict mathematical laws on theories to follow for curve fitting. However, two approaches are generally followed: (1) determine the basic type of function through the study of the physical concepts between the variables and deep understanding of professional knowledge and (2) determine the type of function through the observation of general trends of the curve of experimental data. The root mean square error (RMSE) for the validation data can be calculated for each curve fitting model and used to evaluate the effect of the curve fitting.

Polynomial function models are usually applied to fit a number of kinds of curves. The curve fitting and features extraction can be carried out on not only the adsorption or desorption parts of the response curves respectively, but also the entire response process. In polynomial functions model, *n*-degree polynomials are used to fit the response curves:

(3) $$y=A_{0}+A_{1}x+A_{2}x^{2}+A_{3}x^{3}+\cdots +A_{n}x^{n}$$

During the adsorption part the response curves, *y* represents the sensor response value, *x* represents the time from gas on to gas off. During the desorption part of the response curves, *y* represents the final response value subtracting the sensor value, *x* represents the time from gas off to the end. The fitting model parameters *A_0_*, *A_1_*, *A_2_*, *A_3_*, ···, *A_n_* are used as features.

Exponential function models which are expressed in Equation (4) are another common model in studies used to model E-nose response curves [59]. When the number *N* equals 1 or 2, the exponential function model is called single-exponential function model and double-exponential functions, which are the most commonly used exponential function models:

(4) $$y=A_{0}+\sum_{i}^{N}A_{i}\exp(-\frac{x}{T_{i}}),i=1,2,3,\cdots$$

where *y* and *x* are defined as the same in the polynomial function model and the fitting model parameters *A_0_*, *T_1_*, ···, *T_N_* are used as features.

Auto-Regressive with eXogenous variables (ARX) models are widely built to represent dynamic behavior, used for time series prediction and system identification, and used a different way from the former two types of methods. The combination of sensor and target gas can be regarded as a dynamic system, which gives a response when exposed to a step function. This type of system can be modeled using an ARX model. In [52], not only are simple parameters from original response curves extracted as the features shown in Table 3, but also coefficients from different curve fitting models are taken into consideration. Four models, third-order polynomial function, single-exponential functions, double-exponential functions and ARX model, shown in Table 6, were applied to fit the response curves of E-nose and the corresponding fitting model parameters were extracted as features for recognition.

**Table 6:** Description of features extracted from curve fitting models in [52].

Parameters	Description
Polynomial on/off	$y=A_{0}+A_{1}x+A_{2}x^{2}+A_{3}x^{3}$ On:y=(senor value−baseline) and x=time from gasOn to gasOff Off:y=(maximum value−senor value) and x=time from gasOff to end
Exponential on/off	$y=A\left(1-\exp(-\frac{x}{T})\right)$, where *y* and *x* are defined as in the polynomial fit.
Exponential on/off	$y=A_{0}+A_{1}\exp(-\frac{x}{T_{1}})+A_{2}\exp(-\frac{x}{T_{2}})$, where *y* and *x* are defined as in the polynomial fit.
ARX on/off	$y(t)=a_{1}\cdot y(t-1)+a_{2}\cdot y(t-2)+b\cdot u(t-1)$, On: *y*(*t*) = (sensor value − baseline), *t* time from gas on—5 s to gas off *u*(*t*) = 0 if test gas off and 1 if test gas on Off: *y*(*t*) = (maximum value − sensor value), *t* = time from gas off—5 s to end *u*(*t*) = 1 if test gas off and 0 if test gas on

Table 7 showed neural network prediction results for different features in [52]. As expected, only using one feature, the final response gave a large error. It is possible to improve the prediction accuracies according to the information obtained from the S/σ-ration. On/off derivatives have higher S/a-ratios than max/min derivatives and short on/off integrals, and the prediction errors showed the same tendency than using final responses, so combined with on/off derivatives as features they can obtain lower errors than for the other two combinations (models 2–4 in Table 7). Information from the PCA loading plot can be used to improve the prediction ability too. Final response, 30 s and 90 s on response, which all are adjacent in the loading plot gave worse prediction ability than that of final response together with 30 s and 90 s off response (models 5 and 6 in Table 7), which are located farther. On the whole, the curve fitting model parameters for the entire curves gave worse results compared with the features extracted from original response curves, except for the polynomial coefficients, which can obtain equally good results. Hence, curve fitting parameter features, which refer to the whole response, cannot guarantee better recognition results than those of the simple and piecemeal features combination, which gives comparable results as the more complex curve fitting parameters if chosen carefully.

Carmel *et al.* researched a large dataset composed of 30 volatile odorous substances using a MOSES II E-nose equipped with two sensor modules: an eight-sensor quartz-microbalance (QMB) module and, and an eight-sensor metal-oxide (MOX) module. The samples were put in 20 mL vials in a headspace sampler and the headspace contents were injected into the E-nose, first introduced into the QMB chamber and then the MOX chamber. The injection lasts for 30 s and then a 15 min desorption stage follows. In total, 300 measurements were collected with an average of 10 per chemical. The exponential model, Lorentzian model and double-sigmoid model were applied to fir a curve the response for feature extraction [27].

**Table 7:** Results of neural network estimation from different features in [52].

Model	Features	ANN Architecture	RMSE for Validation (ppm)
1	Final response	1-2-2	13.4
2	Final response	3-3-2	2.2
	On/off derivative		
3	Final response	3-3-2	2.6
	Min/max derivative		
4	Final response	3-3-2	3.6
	Short on/off integral		
5	Final response	3-3-2	4.5
	30 s on response		
	90 s on response		
6	Final response	3-3-2	2.4
	30 s off response		
	90 s off response		
7	Final response	5-4-2	1.6
	30 s on/off response		
	90 s on/off response		
8	Final response	5-4-2	1.7
	On/off derivative		
	on/off integral		
9	Final response	6-5-2	1.7
	On/off derivative		
	Plateau derivative		
	Response/on integral		
10	Polynomial on/off	8-6-2	1.6
11	1. Exponential on/off	4-4-2	2.0
12	2. Exponential on/off	10-6-2	2.1
13	ARX on/off	6-5-2	2.3

The ANN architecture column presents the number of inputs, number of neurons in the hidden layer and number of outputs.

The Lorentzian model is derived from a simple physical description of the measurement process. It applies four parameters, with respective specific physical meaning, which can be obtained from a curve fitting process. The double-sigmoid model was also used for curve fitting. The model with nine parameters function had been constructed by multiplying a monotonically decreasing sigmoid function by a monotonically increasing one. The explicit form of Lorentzian model and double-sigmoid model are expressed in Equations (5) and (6), respectively, and the details of the parameters in the two equations are presented in [27]:

(5) $$x_{i}(t)=\left\{ \begin{array}{l} 0,\;t<t_{i}\\ \beta_{i}\tau_{i}\tan^{-1}\left(\frac{t-t_{i}}{\tau_{i}}\right),\;t_{i}<t<t_{i}+T\\ \beta_{i}\tau_{i}\left[\tan^{-1}\left(\frac{t-t_{i}}{\tau_{i}}\right)-\tan^{-1}\left(\frac{t-t_{i}-T}{\tau_{i}}\right)\right],\;t>t_{i}+T \end{array}\right.$$

where *t* is the response time and t∈[0,T], *T* is the whole response time, *t_i_* is the time how long a particle of the investigated chemical makes its way between the inlet and sensor *i*, which is just the time when the signal starts to rise. The parameters *t_i_*, *T*, $\beta_{i}\text{ and }\tau_{i}$ can be used as features.

(6) $$x_{i}(t)=\frac{\alpha_{i}}{\pi }{\left[1-\exp(-{\left(\frac{t-\beta_{i}}{\gamma_{i}}+e_{i}\right)}^{\delta_{i}})\right]}^{\eta_{i}}\times {\left[\frac{\pi }{2}-\tan^{-1}(\frac{t-\mu_{i}}{v_{i}})\right]}^{\lambda_{i}}$$

where the parameters $\alpha_{i},\beta_{i}\text{, }\gamma_{i}\text{, }e_{i}\text{, }\eta_{i},\mu_{i},v_{i}\text{ and }\lambda_{i}$ can be used as features.

**Table 8:** Classification rates (CR) of different feature sets for QMB or MOX modules in [27].

Classifiers	Modules	Feature Sets	CR (%)
k-Nearest Neighbor (with *k* = 3)	QMB	$maj(\psi^{\max},\beta^{\text{Lor}},\tau^{\text{Lor}},t^{\text{Lor}},T^{\text{Lor}})$	97.8
		$\left(\beta^{\text{Lor}},\tau^{\text{Lor}},T^{\text{Lor}}\right)$	90
		$\left(\psi^{\max}\right)$	84.4
		$maj(\psi^{\max},\beta^{\text{Exp}},\tau^{\text{Exp}},t^{\text{Exp}},T^{\text{Exp}})$	96.7
Mahalanobis distance discrimination in four-dimensional PCA space	QMB	$maj(\psi^{\max},\beta^{\text{Lor}},\tau^{\text{Lor}},t^{\text{Lor}},T^{\text{Lor}})$	83.3
		$\left(\beta^{\text{Lor}},\tau^{\text{Lor}},T^{\text{Lor}}\right)$	96.7
		$\left(\psi^{\max}\right)$	42.2
		$maj(\psi^{\max},\beta^{\text{Exp}},\tau^{\text{Exp}},t^{\text{Exp}},T^{\text{Exp}})$	60
k-Nearest Neighbor (with *k* = 5)	MOX	$maj(\psi^{\max},\beta^{\text{Lor}},\tau^{\text{Lor}},t^{\text{Lor}},T^{\text{Lor}})$	93.1
		$\left(\beta^{\text{Lor}},\tau^{\text{Lor}},T^{\text{Lor}}\right)$	90
		$\left(\psi^{\max}\right)$	86.9
		$maj(\psi^{\max},\beta^{\text{Exp}},\tau^{\text{Exp}},t^{\text{Exp}},T^{\text{Exp}})$	93.5

maj(feature 1,feature 2,...,feature *n*) means the following: use each of the *n* features separately for the purpose of classification, and then decide on the final classification by a majority rule.

Fifty-one feature sets were tested and applied to 101 datasets with nine classifiers. The nine classifiers were based on two types of classifiers. A k-nearest-neighbors (k-NN) classifier [60] with k=1,3,5,7, and classifier in the base of the shortest Mahalanobis distance discrimination [60] in the 1 to 5 dimensional principal components spaces. Average classification performance was calculated and ranked for every feature sets and Table 8 showed the classification performance of several feature sets in [27]. From the ranking of the fifty-one feature sets, the best feature set was $\mathrm{maj}(\psi^{\max},\beta^{\text{Lor}},\tau^{\text{Lor}},t^{\text{Lor}},T^{\text{Lor}})$, which meant classify by majority rule among $\psi^{\max}$, which was the difference between the maximum value and its baseline, and the parameters of the Lorentzian model. The best non-majority feature set was $\left(\beta^{\text{Lor}},\tau^{\text{Lor}},T^{\text{Lor}}\right)$. On the whole, the Lorentzian model was better than the exponential model with respect to classification rate and they were both much better than the standard features, which classified by majority rule among $\psi^{\max}$ and the area beneath the curve, the area beneath the curve left of the peak, and the time it took for the signal to reach its maximum value. Among the three models investigated in [27], the analysis results showed that among the three models (exponential function models, Lorentzian model and double-sigmoid model) with respect to goodness of fit, computation speed, robustness and classification rate, the Lorentzian model was the best model for feature extraction and the most potent set of features resulting in higher classification rates than other combinations of features was $maj(\psi^{\max},\beta^{\text{Lor}},\tau^{\text{Lor}},t^{\text{Lor}},T^{\text{Lor}})$. Some other models are also used to characterize E-nose measurements, such as models based on sigmoid function [61], fractional function, arc tangent function, and hyperbolic tangent function [62]. A summary of different curve fitting models is shown in Table 9.

**Table 9:** Summary of common used curve fitting models.

Model	Description	References
Third-order polynomial function	$Y=A_{0}+A_{1}x+A_{2}x^{2}+A_{3}x^{3}$	[52,62]
Single-exponential function	$Y=A(1-exp(-\frac{x}{T}))$	[52,62]
Double-exponential function	$Y=A_{0}+A_{1}\exp\left(-\frac{x}{T_{1}}\right)+A_{2}\exp\left(-\frac{x}{T_{2}}\right)$	[52,62]
ARX model	$y(t)=a_{1}y(t-1)+a_{2}y(t-2)+bx(t-1)$	[52]
Lorentzian model	Equation (5)	[27]
Double-sigmoid model	Equation (6)	[27]
Sigmoid function	$f(k;\theta )=\theta_{1}\cdot \frac{1}{1+e^{\theta_{2}\cdot (\theta_{3}-k)}}\cdot \left(1-\frac{1}{1+e^{\theta_{4}\cdot (\theta_{5}-k)}}\right)$	[61]
Fractional function	Y=x/Ax+B	[62]
Arctangent function	Y=A×arctan(x/B)	[62]
Hyperbolic tangent function	Y=A×tanh(x/B)	[62]

### 2.3. Features Extraction from Transform Domain

Another method to extract features for E-noses is based on some transforms and then the transform coefficients are used as features to discriminate materials. The most commonly used transforms in E-nose signal processing are the Fourier transform and wavelet transform.

The widely used Fourier transform, for which the basis functions are sine and cosine, maps the original data into a new space. It decomposes the original response into the superposition of the dc component and different harmonic components, and the feature characterized by amplitude of each component can be used for qualitative and quantitative analysis. However, the problem of using the Fourier transform is the impossibility of localizing frequencies in the time domain. This problem can be overcome if we can add the information about the localization of these frequencies in the time domain that can be addressed with the theory of wavelet transform. Wavelet transform [63] is an extension of the Fourier transform. It maps the signals into a new space with basis functions quite localizable in time and frequency space. The wavelet transform decomposes the original response into the approximation (low frequencies) and details (high frequencies). It displays good anti-interference ability [64] for the subsequent pattern recognition if one uses the wavelet coefficients of certain sub-bands as features. Unlike the Fourier transform, the shapes of components of the decomposed signal are different according to the different shapes of the using mother wavelet. The common types of wavelet are Haar wavelet, Daubechies wavelet, Symlet wavelet, Coiflets wavelet, Biorthogonal wavelet, Morlet wavelet, Gaussian wavelet, Mexican hat wavelet, Meyer wavelet, Morlet wavelet, *etc*.

Jia *et al.* [62] constructed a gas sensor array with six metal oxide sensors and one electrochemical sensor and used it to detect the seven species of pathogen most common in wound infections: *P. aeruginosa*, *E. coli*, *Acinetobacter* spp., *S. aureus*, *S. epidermidis*, *K. pneumoniae*, and *S. pyogenes*. They researched and compared the effect of different features of sensors including maximum values, integrals, derivatives, six types of curve fitting model parameters, FFT coefficients and DWT coefficients. In this research, a Daubechies family wavelet (db *N*) was used and the variant *N* was the order of the wavelet. For the Daubechies family wavelet, the *N* was the vanishing moment. A larger *N* can result in stronger localization ability in the frequency domain and better frequency band allocation results, and then, the energy is more concentrated. The results showed that the selected FFT feature, the amplitudes of the DC component, and DWT feature, the mean of the approximating coefficients after decomposition of 12 levels with the sixth order Daubechies family wavelet (db6), can both achieve a 100% correct classification rate, shown in Table 10, which is generally higher than that of the other features except for integral features, single-exponential function and hyperbolic tangent function fitting parameters, which can provide the same 100% correct classification rate.

**Table 10:** Classification rates (CR) of three types of features in [62].

Feature Type	Feature	CR (%)
Original response curve	Maximum	94.29
	Integrals	100
	Derivatives	97.14
Curve fitting parameters	Three-order polynomial function	91.43
	Fractional function	97.14
	Single-exponential function	100
	Double-exponential function	68.57
	Arctangent function	91.43
	Hyperbolic tangent function	100
Transform domain	FFT	100
	DWT	100

Distante *et al.* [50] also analyzed and researched the effect of features of E-noses from original response curves and transform domains for the application to volatile organic compound detection. The results are shown in Table 11. It is evident that the wavelet transform coefficients can obtain the highest classification rate and it is interesting to note that integrals method produces very informative features as compared with the results obtained with the wavelet descriptors.

**Table 11:** Classification rates (CR) of different features in [50].

Feature Type	Feature	CR (%)
Original response curve	Difference maximum	83
	Relative maximum	81
	Fractional maximum	82
	Log maximum	81
	Derivatives	95.45
	Integrals	99.5
Transform domain	Fourier coefficient	96
	Wavelet transform coefficient	100

Ratton *et al*. researched not only the Fourier transform and wavelet transform but also the Gram Schmidt orthogonalization approach in E-nose feature extraction for the investigation of four test gases (methanol, ethanol, formaldehyde, and acetone). For this study, the wavelet transform with Haar wavelet showed the highest overall performance [65]. In [28], a 1-D discrete wavelet transform and a fuzzy adaptive resonant theory map (ARTMAP) neural network are employed as a novel feature extraction and pattern classification method. The result shows that the wavelet technique is more effective than FFT in terms of data compression and is highly tolerant of the presence of additive noise and drift in the sensor responses. Tian *et al*. [32] proposed a new method of detecting wound pathogens by selecting the wavelet transform coefficients preferentially with a scatter matrix and using the mean of the selected coefficients as the feature. The new feature extraction method showed high performance in identifying seven species of pathogens. Without the influence of drift, the classification rate of the new features using a probabilistic neural network classifier can reached 100% while the maximum value feature obtained only 88.57%. Under the effect of a strong drift, although both the classification rates decreased, the anti-drift ability of the new features was evidently stronger than that of the maximum value feature. Ionescu *et al.* [66] demonstrated that a single, thermally modulated tungsten oxide-based resistive sensor can discriminate between different vapours based on DWT. It can be found that DWT outperformed FFT in the extraction of important feature from the sensor response and allowed for straightforward gas recognition in feature space according to the 2-dimension PCA score plots.

### 2.4. Feature Extraction from Phase Space

The phase space (PS), which can be used for signal processing in many fields [67,68,69], is a key concept in the field of dynamic systems research. We assume that the state of a system is completely described by *m* scalar variables. The different states are represented as different points in an *m*-dimension vector space defined by an orthonormal basis where each direction is corresponding to one of the scalar variables. The basic characteristic of the PS is the correspondence between each point and the transient state of the system. A general PS can be defined according to Taken’s embedding theorem [70]. In PS, the time course of any system is described by time parametric trajectories, which contains the dynamic characteristics of the system. The trajectories present a large variety of shapes depending on the nature of the phenomena. Neglecting the scale effects, the shapes of the trajectories are expected to be associated to the properties of the phenomena. From this point of view, it is interesting to define some morphological descriptors which are able to encode the shape of the trajectories. These morphological descriptors can then be used to obtain information about the system dynamics and used as the features [71].

**Figure 1:**
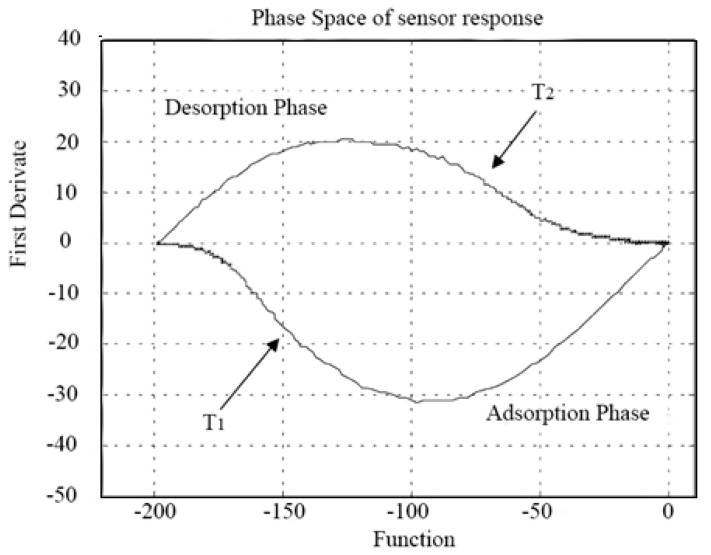
Phase space of sensor with f(t) and f′(t) as variables.

The first attempts to introduce the PS and dynamic moments (DM) [72,73] to represent the temporal evolution of chemical sensor signals (QCM) was presented in Refs. [33,34]. Martinelli *et al*. described the sensor signal changes in a PS by the response values and its first derivative like Figure 1 and then extracted the area, called phase space integral (PSI), spanned by the trajectory during the evolution either in adsorption or in desorption phase as feature, which gave a substantial improvement in terms of both error of estimation and classification [33].

Vergara *et al.* took some morphological descriptors DM in PS analogous to the second moments of the area of a geometrical figure in a 2D space into consideration. The usefulness of the method of PS and DM is assessed by analyzing the transient response of metal oxide gas sensors either to a step change in gas concentration or to thermal modulation [71].

**Figure 2:**
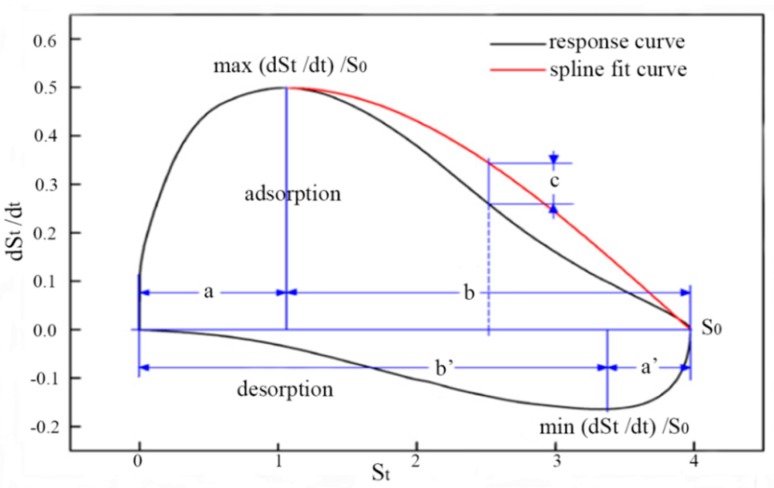
Six parameters extracted from PS.

In [74], a gas sensor array with six TGS sensors, which were TGS813, TGS2600, TGS2602, TGS2610, TGS2611 and TGS2620, was used in the investigation on 11 kinds of gases at four different concentrations. The test gases were benzene, toluene, xylene, acetone, butanone, methanol, ethanol, formaldehyde, acetaldehyde, pentane and cyclohexane, while the four concentrations were 100, 200, 300 and 400 ppm. The phase space entire (PSE) feature extraction method extracted six features from PS (Figure 2), which can describe the response curves completely. Suppose $S_{t}$ to be the response of sensors, which is a function of time. Six parameters which can be obtained from the response curve in PS are $S_{0}$ (the maximum of $S_{t}$), max$\left(dS_{t}/dt\right)/S_{0}$ (the ratio of height and length in adsorption process), a/b (the location of ma$\left(dS_{t}/dt\right)$), min$\left(dS_{t}/dt\right)/S_{0}$(the ratio of height and length in desorption process), $a^{\prime }/b^{\prime }$(the location of min$\left(dS_{t}/dt\right)$) and *c* is wrap value, which details are expressed in [74]. The difference of PSE from other methods based on PS is that the sensor response curves could be reconstructed well with the six features in PSE. PSE was compared with the PSI and maximum value methods and the recognition results of three feature methods with FDA are shown in Table 12.

**Table 12:** Classification rates (CR) of different features in [74].

Features	Number of Features	CR (%)
Relative difference maximum	1	90.9
Phase space integral (PSI)	1	81.1
Phase space entire (PSE)	6	100

### 2.5. Other Methods for Feature Extraction

Besides the above feature extraction methods, many new original methods have been proposed in recent years. The “fingerprint” obtained from an E-nose is strongly dependent on the kind and concentration of volatiles to which it are exposed, the duration of this exposure and the temperature and humidity of the system. In some cases, it can be appropriate to increase the amount of data in order to capture as much information as possible which properly describes the system, if more reliable models are desired.

A novel method of feature extraction is the use of the three-way structure of the signals obtained from the recording of the different sensor values during a period of time, for each sample. Following this strategy, the contribution of the inner relationship between the three ways can be exploited to obtain more robust information about the system. Parallel factor analysis (PARAFAC) is one of the most popular multi-way data decomposition methods. Originated from psychometrics [75,76], PARAFAC is gaining interest because it is a processing technique which simultaneously determines the pure contributions to the dataset, optimizing each factor as a time, in trilinear systems. In [35], the use of PARAFAC as a feature extraction technique from a specific three-way dataset (samples × sensors × time) for food investigation by E-noses was researched. PARAFAC performed a suitable feature extraction task, incorporating the most of the information of this complex data system. The methodology of PARAFAC also was applied in feature extraction of gas sensor data of monitoring the indoor air pollutants. The results showed that PARAFAC could more accurately monitor and predict the indoor air quality variations than multiway principal component analysis (MPCA), since it is able to capture both the time-correlations and the variable-correlation between indoor air pollutants [36]. 

Due to the fact that the sensitivities of the gas sensors are different and the signals of the measured samples also are different, the energy characteristics of the signals can be used for feature extraction. An approach called global feature extraction, which focuses on the whole sensors array instead of individual sensors, based on the mutual energy of sensor signal called energy vector (EV) was introduced by Vergara [37]. It is very useful to study the behavior of individual sensors and the theory of signals about the mutual energy is suitable to study the relations between signals of sensors belonging to the same array. The sensors of an array simultaneously interact with the same chemical pattern and as a consequence the sensor signals share a certain degree of correlation. The amount of correlation may change according to the quality and quantity of the materials to which the array is exposed. The EV was defined as:

(7) $$E=\left[\xi_{1,1},\xi_{1,2},...,\xi_{1,n},\xi_{2,1},\xi_{2,2},...,\xi_{2,n},...,\xi_{n-1,n-1},...,\xi_{n,n}\right]$$

where $\xi_{xy}=\int_{t_{0}}^{t_{1}}x(t)y(t)dt$ was the mutual energy between signals *x*(*t*) and *y*(*t*) in the interval (*t*_0_, *t*_1_) and *n* is the number of sensors that compose the array. The method was assessed by solving a practical problem of identification and prediction of pollutant species by building and validating a linear discriminant analysis classifier method performed by partial least squares (PLS-DA). Different validation techniques have been implemented, which have shown that the EV provides sufficient accuracy, and then it is of interest for simple, small size and real time gas analyzers. The results showed that this feature extraction methods based on EV outperformed the FFT method [37].

Kish *et al.* presented a new feature extraction method for E-noses based on a resistance noise power density spectrum (PDS), which demonstrates that even a single sensor can be sufficient to distinguish between many different chemical species [38]. In this method, it is first assumed that only one sensor is used and its response is independent for each investigated gas. If the PDS of the resistance fluctuations in the sensor has K different frequency ranges, in which the dependence of the response on the concentration of the gases is different from the response in the other ranges, one can write:

(8) $$\begin{array}{l} dS\left(f_{1}\right)=B_{1,1}C_{1}+B_{1,2}C_{2}+\cdots +B_{1,N}C_{N}\\ \vdots \\ dS\left(f_{K}\right)=B_{K,1}C_{1}+B_{K,2}C_{2}+\cdots +B_{K,N}C_{N} \end{array}$$

where *dS*(*f*_i_) is the change of the PDS of resistance fluctuations at the *i*-th characteristic frequency (or frequency range), and the *B_i,j_* quantities are calibration constants in the linear response. Thus, a single sensor is able to provide a set of independent equations to determine the gas composition around the sensor. If we have *P* different sensors, and if we can use the same characteristic frequency ranges for all sensors, then we have:

(9) $$\begin{array}{l} dS^{\left(1\right)}\left(f_{1}\right)=B_{1,1}C_{1}+B_{1,2}C_{2}+\cdots +B_{1,N}C_{N}\\ \vdots \\ dS^{\left(1\right)}\left(f_{K}\right)=B_{K,1}C_{1}+B_{K,2}C_{2}+\cdots +B_{K,N}C_{N}\\ dS^{\left(2\right)}\left(f_{1}\right)=B_{1,1}C_{1}+B_{1,2}C_{2}+\cdots +B_{1,N}C_{N}\\ \vdots \\ dS^{\left(2\right)}\left(f_{K}\right)=B_{K,1}C_{1}+B_{K,2}C_{2}+\cdots +B_{K,N}C_{N}\\ \vdots \\ dS^{\left(P\right)}\left(f_{K}\right)=B_{K,1}C_{1}+B_{K,2}C_{2}+\cdots +B_{K,N}C_{N} \end{array}$$

where *dS*^(m)^(*f*_i_) is the change of the PDS of resistance fluctuations at the *i*-th characteristic frequency range in the *m*-th sensor and the number of independent equations is P×K.

Wang *et al.* compared three E-nose features for tomato detection: kurtosis coefficient features, similitude entropy features and the energy features. The results showed that according to the separability measurement, the similitude entropy feature extraction method had its advantages in electronic nose detection [77].

**Figure 3:**
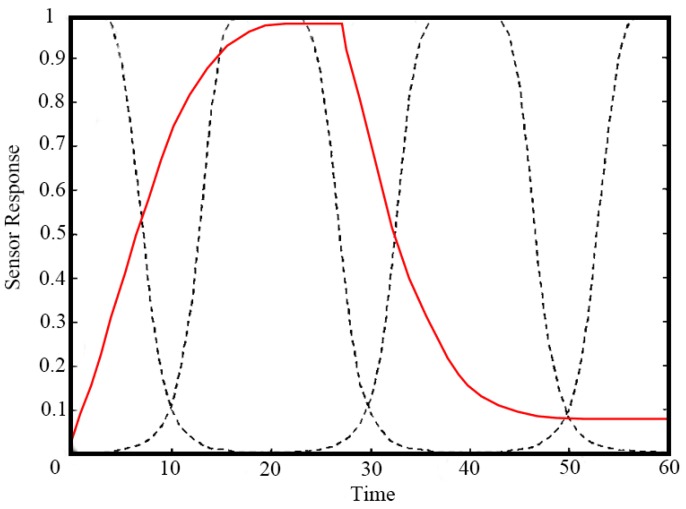
Temporal representation of WTS.

Gutierrez-Osuna *et al.* [39] suggested a method called window time slicing (WTS) for creating pseudosensors based upon time slicing of sensor responses, wherein, the time response of each sensor was multiplied by four time windows to obtain the area values shown in Figure 3. First, the response signals are multiplied by four smooth, bell-shaped windowing functions as shown in Figure 3, and then the resulting time traces are integrated with respect to time as shown in Equation (10). In fact, the integrals are the areas surrounded by the response signal curves and the four windowing function curves. Therefore, for each sensor, four “areas” are obtained, which represent the area under the two curves. The windows are designed to overlap and span the entire response interval. These areas were further used as features and were selected using GA for classification of fragrances, hog farm air and cola beverage samples [78]:

(10) $$\begin{array}{l} A=\left[W^{1},W^{2},\ldots ,W^{4}\right]\\ W^{i}=\sum_{k=1}^{N}R\left(t_{k}\right)K_{i}\left(t_{k}\right)\Delta t\\ K_{i}\left(t_{k}\right)=\frac{1}{1+{\left(\frac{t_{k}-c_{i}}{a_{i}}\right)}^{2b_{i}}} \end{array}$$

where *A* is the feature vector extracted from one sensor which consist of four elements, $W^{i}$ is the area surrounded by the response signal curve and the windowing function curves, $R\left(t_{k}\right)$ is the response value at the time $t_{k}$, $K_{i}\left(t_{k}\right)$ is the value of the *i*-th windowing function at the time $t_{k}$, k=1,2,…,N denotes the sampling points of each curve, Δt is the sampling interval between two sampling points, and the parameters $\left(a_{i},b_{i},c_{i}\right)$ define the width, shape and center of the different windowing functions $K_{i}\left(t_{k}\right)$.

**Figure 4:**
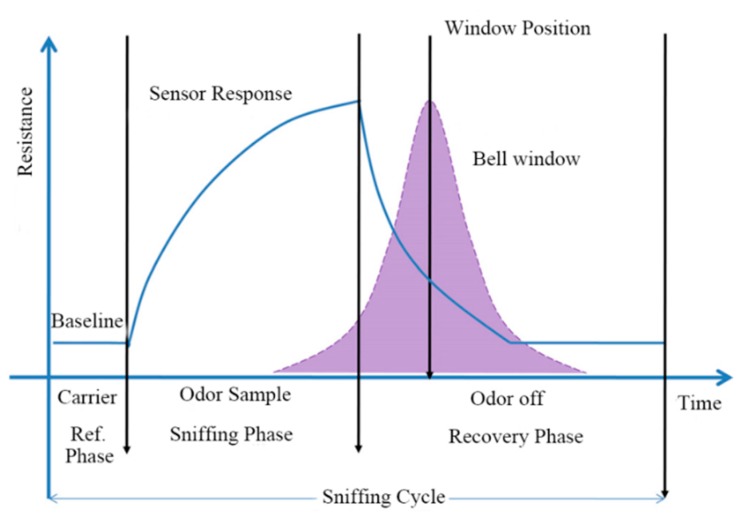
Schematic diagram of MWTS technique.

However, the drawback of this method is the loss of information owing to the gap between each window. In [79], an attempt has been made to identify the optimum positions of the time windows in order to maximize the classification rate. To avoid the drawback of WTS, a bell shaped window defined by normal probability distribution function with mean at its position and standard deviation as 10 s was moved along the time response (shown in Figure 4) of the sensors and simultaneously multiplied with the sensor response to obtain the area values at different positions. It can be proved that moving window time slicing (MWTS) as an approach to extract area values as features can enhance the performance of an E-nose [80]. Comparing MWTS with other techniques *viz*. peak value, rise time, fall time, DWT, FFT and WTS, it can be seen that, in Table 13, the MWTS method performed better than the others for classification of Kangra orthodox black tea.

**Table 13:** Classification rates (CR, %) of different features in [80].

Features	All Sensors	Sensors 2–4	Sensors 2 and 3	Sensors 2 and 4	Sensors 3 and 4
Maximum value	72.95	75.72	75.22	74.9	74.83
Rise time	75.5	53.43	41.25	34.95	36.18
Fall time	61.88	87.70	85.12	83.17	93.43
DWT	86.32	94.67	95.07	94.70	94.90
FFT	84.70	92.17	92.05	92.02	91.97
WTS	89.40	92.92	92.54	92.02	93.20
SITO-WTS ^a^	–	–	–	–	94.52
SITO-MWTS ^b^	–	97.35	96.20	93.30	96.35

^a^ Considering position 4 of sensor 3, and positions 1 and 2 of sensor; ^b^ Considering bin 3 of sensors 2 and 4, and bin 10 of sensor 3.

**Figure 5:**
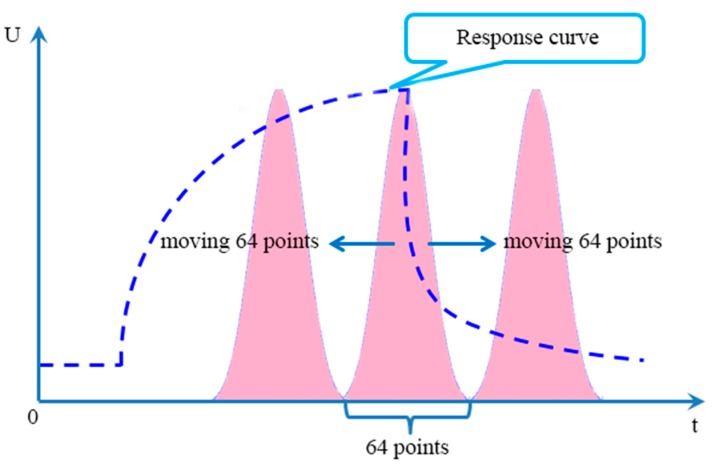
The schematic diagram of MWFC.

Guo *et al.* [58] proposed a novel feature extraction method also based on window functions called moving window function capturing (MWFC) shown in Figure 5. A 64 points window was placed around the peak value and then moved 64 points to the left and right along with the time axis, respectively. Thus three area values surrounded by two curves can be obtained during the moving process and the three area values were chosen as features simultaneously. The width, position, shape of the window were compared and discussed, and the final results showed that the MWFC feature performed better than the contrast features such as peak value, rising slope, descending slope, FFT, DWT and WFC, showing in Table 14. The same conclusion can be drawn for another two E-nose datasets [81,82].

**Table 14:** Classification rates (CR) of different features in [58].

Feature Extraction	Accuracy Rate
Peak value	87.5
Rising slope	87.5
Descending slope	85
FFT	90.0
DWT	92.5
WFC	95.0
MWFC	97.5

## 3. Conclusions and Outlook

E-noses have been utilized in a wide and diverse range of applications such as odor analysis, quality control in the food industry, environmental protection detection, public health, explosives detection and spaceflight applications to improve effectiveness, efficiency and safety. Due to the complexity of biological olfaction, the artificial olfactory system has non-linearity characteristics. The characteristic response of the sensor array is a whole cognition of the odors called “fingerprint”. It is crucial to extract useful and robust information from the sensor characteristic response with less redundancy. This paper has presented an overview of the methods of feature extraction used in E-noses for different applications in recent years, such as extraction from the original response curves, curve fitting parameters, transform domains, phase space and dynamic moments, parallel factor analysis, energy vector, power density spectrum and window functions, *etc*. A few additional approaches, not covered in this review due to space constraints, also offer promising results. Feature extraction from the sensor signals is a key procedure to further improve the performance of an E-nose, but the evaluation of a feature extraction method is influenced by the type of sensors, parameters of experiments, detection targets, demands of specific application, and so on. Different methods are suitable for different situations, and we have to choose the method according to the actual conditions. Although there are no widely approved evaluation criteria for various features, we can give some advice on feature extraction according to the previous research. On the whole, for steady-state features, normalization models give better performance than other models. Transient features have more information than steady-state features. Integrals usually give better performance than maximum values, and the same conclusion can be drawn for derivatives, especially when one sensor extracts several derivatives. Curve fitting parameter features cannot guarantee better recognition results than those of the simple and piecemeal features combination, which gives comparable results to more complex curve fitting parameters if chosen carefully. The single-exponential model is a fine choice, which is simple and easy to deploy and can usually obtain better performance than steady-state features. DWT generally obtains better results than any other feature extraction. In addition, we also hope that more effective feature extraction strategies such as feature fusion can be proposed in further work for enhance the performance of E-noses with the inspiration provided in this paper. Due to the differences of selectivity, sensitivity and specificity of sensors, the optimal features of sensors are various. Usually, the same features are extracted from different sensors with the special methods which we reviewed above. However, it is possible that the features are not optimal for each sensor and better performance can be obtained if different features are extracted and fused for pattern recognition with different feature methods for different sensors.
